# TRAIL as a Warrior in Nano-Sized Trojan Horse: Anticancer and Anti-Metastatic Effects of Nano-Formulations of TRAIL in Cell Culture and Animal Model Studies

**DOI:** 10.3390/medicina60121977

**Published:** 2024-12-01

**Authors:** Ammad Ahmad Farooqi, Assiya Turgambayeva, Gulnara Kamalbekova, Roza Suleimenova, Natalya Latypova, Sholpan Ospanova, Dinara Ospanova, Zhanat Abdikadyr, Sabit Zhussupov

**Affiliations:** 1 Department of Molecular Oncology, Institute of Biomedical and Genetic Engineering (IBGE), Islamabad 44090, Pakistan; 2 Department of Public Health and Management, Astana Medical University, Astana 010000, Kazakhstan; assiya739@gmail.com (A.T.); ospanova.sh.k@gmail.com (S.O.); 3 Department of Family Medicine, Astana Medical University, Astana 010000, Kazakhstan; kamalbekowa@gmail.com (G.K.); nata77ru@yandex.kz (N.L.); 4 Department of Public Health and Hygiene, Astana Medical University, Astana 010000, Kazakhstan; suleimenova.r@amu.kz; 5 Faculty of Medicine and Healthcare, Al-Farabi Kazakh National University, 71 Al-Farabi Ave, Almaty 050040, Kazakhstan; dinara.ospanova.alm@gmail.com; 6 Department of Biostatistics, Bioinformatics and Information Technologies, Astana Medical University, Astana 010000, Kazakhstan; zhanna.abdikadyr@gmail.com; 7 Department of Surgery, Semey Medical University, Semey 071400, Kazakhstan; sabit.73@mail.ru

**Keywords:** TRAIL, apoptosis, cell signaling, metastasis, xenografted mice, nanotechnology, nano-formulations

## Abstract

Cancer is a therapeutically challenging and genomically complicated disease. Pioneering studies have uncovered multifaceted aspects of cancer, ranging from intra- and inter-tumor heterogeneity, drug resistance, and genetic/epigenetic mutations. Loss of apoptosis is another critical aspect that makes cancer cells resistant to death. A substantial fraction of mechanistic information gleaned from cutting-edge studies has enabled researchers to develop near-to-complete resolution of the apoptotic pathway. Within the exciting frontiers of apoptosis, TRAIL (tumor necrosis factor-related apoptosis-inducing ligand) has garnered phenomenal appreciation by interdisciplinary researchers principally because of its unique capability to target cancer cells. TRAIL-based monotherapies and combinatorial therapies have reached phase II and phase III clinical trials. Rapidly upgrading the list of clinical trials substantiates the clinically valuable role of TRAIL-based therapeutics in cancer therapy. However, there is a growing concern about the poor bioavailability and rapid clearance of TRAIL-based therapeutics. Excitingly, the charismatic field of nanotechnology offers solutions for different problems, and we have witnessed remarkable breakthroughs in the efficacy of TRAIL-based therapeutics using nanotechnological approaches. In this review, we have attempted to provide a summary about different nanotechnologically assisted delivery methods for TRAIL-based therapeutics in cell culture studies and animal model studies for the inhibition/prevention of cancer.

## 1. Introduction

High-throughput technologies have generated comprehensive data across various omics layers and offered previously unprecedented insights into the molecular intricacies of carcinogenesis and metastasis. A wealth of information has enabled researchers and clinicians to develop a better understanding of the complex choreography of cell intrinsic and extrinsic factors that underlie carcinogenesis and metastasis. Accordingly, high mutational burden, deregulation of cell signaling cascades, loss of apoptosis, and drug resistance play a contributory role in the onset and progression of cancer [1,2,3].

Increasingly, it is being recognized that metastatic cancers remain incurable primarily because of the emergence of apoptosis-resistant clones, tumor evolution, and intra-tumor heterogeneities. Improvement in the efficacy of the treatment outcome not only depends on the highly selective ability of anticancer drugs to kill cancer cells but also on a robust activation of the immune system against the tumors for the eradication of residual cancer cells that may survive treatment [4,5]. For the elimination and eradication of malignant solid tumors, both lymphoid and myeloid hematopoietic cells extravasate from the bloodstream into the tumor tissues. Later, they migrate to specific niches of the tumor microenvironment for a functional interaction with each other, with non-hematopoietic stromal cells, and eventually, with cancer cells. Intriguingly, these interactions modulate proliferative expansion, differentiation, and the execution of antitumor or pro-tumor effector functions, which holistically shape the outcome of therapeutically induced or spontaneous antitumor immunological responses [6,7].

**Figure 1:**
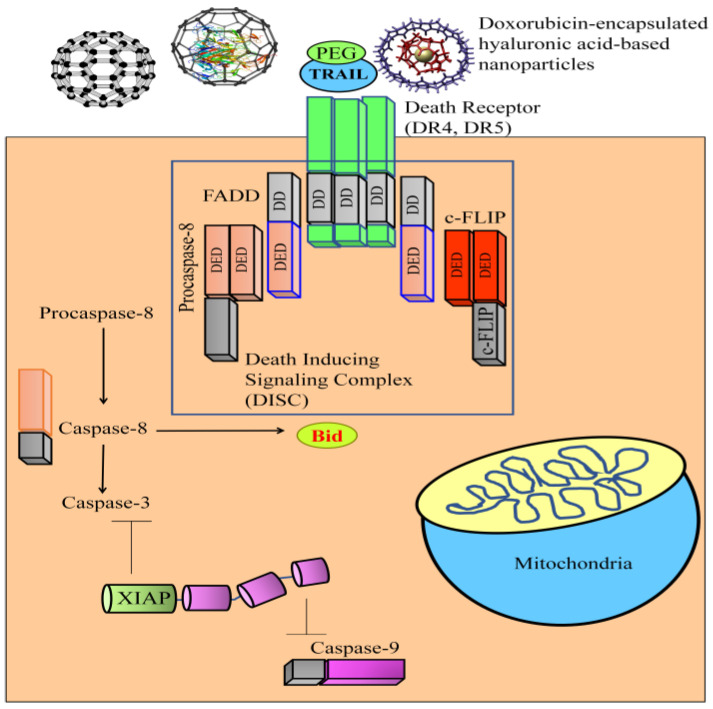
TRAIL-mediated intracellular signaling induced the activation of caspase-8 and caspase-3. Different nano-formulations have been designed to improve the efficacy and bioavailability of TRAIL-based therapeutics. TRAIL exerted apoptotic effects through two characteristically distinct pathways (extrinsic and intrinsic). Multi-protein complex (DISC) formed at death receptor (DR4 and DR5) induces the activation of caspase-8. Caspase-8 has been interconnected with intrinsic pathway through proteolytic processing of Bid protein. Truncated Bid moves into the nucleus and triggers the release of cytochrome c and various other proteins. Cytochrome c induces the assembly of adaptor protein complex (apoptosome) for the activation of caspase-9. XIAP is an anti-apoptotic protein having a unique ability to inhibit caspase-9 and caspase-3. PEGylated TRAIL works efficiently in combination with chemotherapeutic drugs in the induction of apoptotic death in TRAIL-resistant cancer cells.

Loss of apoptosis is a major cause of cancer onset and progression [8,9,10,11]. Research during the 20th century has shown the existence of a superfamily of tumor necrosis factor (TNF) proteins. TNF was only capable of inducing necrosis in selected cancers. Moreover, systemic administration of recombinant TNF induced lethal inflammation. Later, biochemists started to analyze the therapeutic value of FasL (CD95L). However, FasL demonstrated high systemic toxicity. TRAIL (tumor necrosis factor-related apoptosis-inducing ligand) has captured extraordinary appreciation because of its hallmark feature to target cancer cells while sparing the normal cells. TRAIL transduces the signals intracellularly through death receptors. DR4 (death receptor 4) and DR5 (death receptor 5) are involved in the transduction of the signals by formation of a DISC (death-inducible signaling complex). Selective targeting of cancer cells makes TRAIL-mediated signaling characteristically and functionally superior in cancer biology. Accordingly, we have witnessed breakneck growth in the pioneering research works related to TRAIL-mediated signaling in different cancers. In the past two decades, proof-of-concept studies have comprehensively characterized the signaling machinery of the TRAIL/death receptor pathway in various cancers [12,13]. However, contemporary studies also highlighted TRAIL-resistant cancer cell lines, which urged researchers to re-interpret the underlying causes of TRAIL resistance. Low expression of death receptors on the surface of cancer cells led to significant impairment of the TRAIL-mediated apoptotic pathway [14,15]. Therefore, medicinal chemists overcome the stumbling block by stimulating the surface expression of death receptors by treatment with natural products and synthetic agents. Natural products re-sensitized cancer cells to TRAIL-mediated apoptotic death via upregulation of death receptors, overexpression of pro-apoptotic proteins, and inactivation of anti-apoptotic proteins. TRAIL-mediated intracellular signaling induced the activation of caspase-8 and caspase-3 (Figure 1). TRAIL-based therapeutics have been tested in combination with chemotherapeutics and TRAIL-sensitizers. The rationale is that cancer cells gradually develop resistance against TRAIL-based therapeutics by inhibition of the extrinsic and intrinsic pathways. In addition, c-FLIP has been reported to interfere with the formation of DISC (death-inducible signaling complex) by competing with caspase-8 for binding to FADD. Likewise, XIAP interacts with and inhibits caspases, and its frequent overexpression leads to TRAIL resistance. SMAC mimetics have been developed to impair the functions of XIAP (Figure 1). Gaze through the scientific lens suggested that mechanistic dissection of signaling machinery of the TRAIL pathway paved the way for the design and development of TRAIL-based therapeutics. 

Nanotechnology has strategically empowered interdisciplinary researchers to overcome the stumbling blocks associated with conventional deliveries. These bottlenecks include large-scale issues such as biodistribution to smaller-scale barriers such as sophisticated intracellular transportation. Therefore, specific targeting and smartly designed nano-materials for the delivery of payloads through molecular transportation to specified organelles is a rapidly growing dimension of nanotechnological research [16,17,18,19,20]. For realistic analysis of clinical translatability of future-oriented and exciting nano-enabled technologies, in 2000, the US National Science and Technology Council launched NNI (National Nanotechnology Initiative). The main purpose was to identify grand challenges and well-defined initiatives for the advancements in the field.

Poor bioavailability of TRAIL-based therapeutics is a rapidly growing concern. To overcome these bottlenecks, interdisciplinary researchers decided to reap the benefits of nanotechnological advancements. We have witnessed an exponential increase in the methodology related to the nanotechnological delivery of TRAIL. Encapsulation of TRAIL inside the particles resulted in a sustained and constant release from the particles. Moreover, attachment of TRAIL to the surface of nanoparticles allows immobilization of TRAIL, thus mimicking a membrane-bound protein. Use of nanotechnological strategy for the delivery of TRAIL-based therapeutics has revolutionized the field of molecular oncology. In this mini-review, we have gathered some of the most promising nanotechnological breakthroughs that have considerably improved and enhanced the delivery of TRAIL to the target sites in tumor-bearing mice.

## 2. Bioavailability of TRAIL

Poor systemic distribution of TRAIL administration has urged researchers to explore different opportunities for efficient distribution of TRAIL-based therapeutics to the target sites.

Structurally, native TRAIL exists as a noncovalently associated trimer (~60 kDa). In the kidney, it gets dissociated into monomers (~20 kDa), thus resulting in a rapid clearance. Importantly, its clearance rate was similar to the predicted clearance rate for 20 kDa proteins, which provided the underlying causes for the failure of dulanermin in phase II trials. Poor pharmacokinetic properties of dulanermin because of its smaller molecular weight led to rapid elimination via renal filtration. Dulanermin had a distribution half-life of only 3–5 min and an elimination half-life of 20 min [21,22]. The addition of a molecular tag increases the size and improves its half-life. TRAIL therapeutics were strategically improved by the addition of a specific trimerization domain, a modified leucine zipper motif (LZ-TRAIL), and isoleucine zipper (iz-TRAIL) at the N-terminus of the extracellular domain. Interestingly, these trimerization motifs allowed the researchers to achieve exceptional stability of the TRAIL trimers because of interactions between isoleucine or leucine zipper domains that formed stable triple helices. Therefore, recombinant forms of TRAIL demonstrated superior pharmacokinetic profiles with a prolonged distribution half-life of 1.3 h and an elimination half-life of 4.8 h.

TLY012, an N-terminal PEGylated rhTRAIL, has been designed to improve the half-life and reduce the clearance by renal filtration. INBRX-109 is another multivalent DR5 agonistic antibody. Previous findings suggested that multivalent antibodies led to unexpected, significant but reversible hepatotoxicity (as evidenced by the termination of the phase 1 trial for TAS266, a DR5-targeting tetravalent nanobody). In accordance with the previous results, INBRX-109 was engineered to avoid hepatotoxicity by limiting its valency to four.

A team of researchers did not observe secretion of any intact TRAIL in the urine, and a high amount of radioactivity in the urine most likely reflected degradation products of TRAIL [23].

Since delivery of therapeutic drugs is extremely challenging because of the blood–brain barrier (BBB) in brain tumors, delivery of the anticancer agents into tumor tissues is indeed a valuable approach for the clinical treatment of brain tumors.

Convection-enhanced delivery of TRAIL resulted in the distribution of almost the entire tumor and portions of surrounding normal tissues, suggesting dense distribution within the tumor and tumor margins by TRAIL [24].

## 3. Delivery of TRAIL Protein

**Figure 2:**
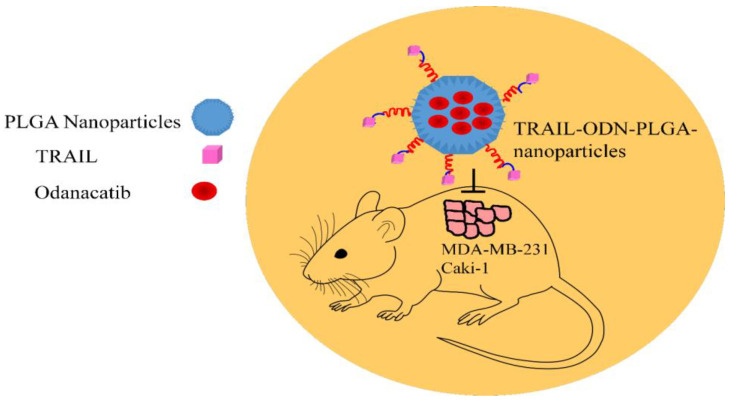
Odanacatib (ODN) loaded poly (lactic-co-glycolic) nanoparticles conjugated to TRAIL inhibited tumor development in mice.

Odanacatib is a potent and selective cathepsin K inhibitor for cancer treatment. Odanacatib (ODN)-loaded poly(lactic-co-glycolic) nanoparticles conjugated to TRAIL were found to be effective against TRAIL-resistant cancers. Odanacatib sensitized cells to anticancer drugs through suppression of RAPTOR and p53-dependent activation of BAX. TRAIL-ODN-PLGA nanoparticles efficiently induced shrinkage of the tumor xenografts derived from MDA-MB-231 and Caki-1 cancer cells in animal models (Figure 2) [25].

**Figure 3:**
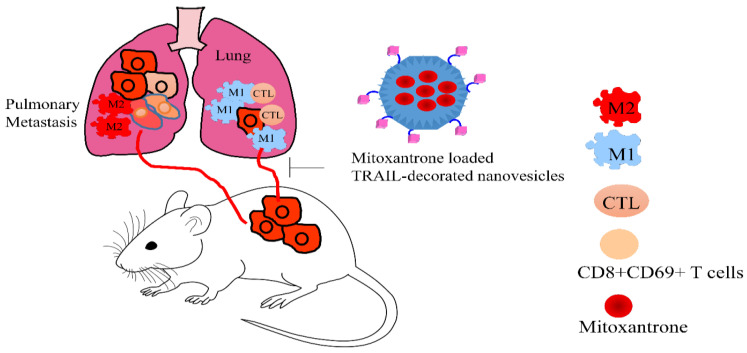
Mitoxantrone loaded TRAIL-decorated nanovesicles inhibited metastatic dissemination of cancer cells and re-shape tumor microenvironment. These nanovesicles promoted the re-polarization of macrophages from M2 to M1 phenotype. CD8 T cells and CTL were found to be enhanced within tumor microenvironment for the eradication of the tumors.

Mitoxantrone combined with engineered TRAIL nanovesicles induced pyroptosis in cancer cells and inhibited the tumor growth and metastasis. The microparticles released by pyroptotic tumor cells also demonstrated cytotoxic effects against other tumor cells. In the tumor microenvironment, tumor-associated macrophages exist in an immunosuppressive M2-like phenotype. However, it has been shown that cell pyroptosis caused re-polarization of the M2-like phenotype to the M1-like phenotype. Dendritic cells promoted the activation and infiltration of T cells and potentiated antitumor immunological responses. The proliferation and activity of CTL (CD3+CD8+) and CD3+CD8+CD69+ T cells were found to be enhanced in mice treated with mitoxantrone-loaded TRAIL-decorated nanovesicles. Collectively, these nanovesicles also potently impaired the metastatic dissemination of cancer cells to the lungs (Figure 3) [26].

DOTAP-mPEG-PCL (DMC) nanomicelles served as an efficient delivery system capable of forming nano-complexes with siRNAs. ERCC2 overexpression in LN229 and U87 cells increased proliferative and colony-forming abilities of cancer cells. There was an accelerated tumor growth and larger tumor weight in mice inoculated with ERCC2 overexpression-GL261 cells. siERCC2-loaded DMC nano-complexes were injected intratumorally in combination with TRAIL and interfered with the growth of the tumors derived from U87 glioblastoma cells [27].

In another study, mixing positively charged TRAIL with hyaluronic acid (HA) resulted in the formation of functionally efficient nanocomplexes. Findings indicated that the complex formation did not interfere with the TRAIL activity. Additionally, TRAIL stability in these complexes was retained for 6 days, whereas the bioactivity of native TRAIL was noted to be reduced. Moreover, plasma concentration levels of subcutaneous injections of native TRAIL were not detectable after 12 h in the rats. However, a substantial amount of TRAIL was detected in blood for up to 5 days in rats subcutaneously injected with HA/TRAIL nanocomplexes [28].

Doxorubicin was encapsulated into the micelles, and TRAIL was decorated on the surface of cationic micelles as a promising delivery agent for the combinatorial delivery of chemotherapeutic drugs and TRAIL [29]. Paclitaxel is loaded into the core of the micelles, and the surface of these micelles is modified with TRAIL. Combinatorial delivery of TRAIL and paclitaxel using nanoparticles is valuable as it reduces the number of administrations and simultaneously delivers both TRAIL and paclitaxel to cancer cells [30]. These delivery vehicles can be tested for efficiency in tumor-bearing mice and metastasis models.

Doxorubicin-loaded human serum albumin nanoparticles surface-modified with TRAIL and transferrin are efficient against different cancers. It was shown that tail vein injections of TRAIL/Tf/Dox-loaded nanoparticles inhibited the growth of the tumors in mice inoculated with HCT 116 cells. TRAIL/Tf/Dox-loaded nanoparticles were maximally distributed within tumor sites, and their retention was maintained with only a slight decrease until 32 h post-injection [31].

Dacarbazine, a chemotherapeutic drug, has been found to be effective against different cancers. Dacarbazine-loaded polylactic acid (PLA) nanoparticles are conjugated covalently to a specific DR5-targeting monoclonal antibody. These drug-loaded nanoparticles conjugated to DR5-targeting antibodies were found to be effective against melanoma cells. Expectedly, DR5 antibody-modified nanoparticles demonstrated robust binding affinities for A375 (DR5-overexpressing) cells, but these nanoparticles did not display significant binding affinity for DR5-negative cells [32].

PEGylated heparin and poly-l-lysine (PLL) were used for the preparation of TRAIL-loaded nanoparticles. Accordingly, the half-life of TRAIL in PEG-NPs was 28.3-fold higher as compared to TRAIL alone. One hundred micrograms of intravenously administered TRAIL-PEG-NPs every four days induced shrinkage of tumors in BALB/c athymic mice subcutaneously implanted with HCT-116. Histologically analyzed liver sections did not show TRAIL-PEG-NP-mediated pathological effects even at day 24 [33].

TRAIL-loaded human serum albumin (HSA) nanoparticles have been tested for pharmacokinetic properties and anticancer effects. Intravenously administered TRAIL-loaded human serum albumin (HSA) nanoparticles in male Sprague Dawley rats demonstrated sustained release, whereas TRAIL was rapidly eliminated. There is an increase in the half-life (9.2-fold) of TRAIL-HSA nanoparticles. Whereas systemic clearance of TRAIL-loaded HSA was found to be reduced up to 9.2-fold as compared to TRAIL. Concentration levels of TRAIL within tumor tissues were markedly high in rodent models administered with TRAIL-HSA nanoparticles. Importantly, accumulation of TRAIL-HSA-NPs in the tumors was enhanced potently at 1 h after injection. Notably, the accumulation rate of TRAIL-HSA nanoparticles was about 14-fold (1 h point) as compared to TRAIL. Moreover, at 4 h after dosing, distribution of TRAIL-HSA-NPs within tumor tissues was still found to be significantly high [34].

Functionalization of natural killer (NK) cells with liposomes conjugated with TRAIL and an antibody against the NK1.1 antigen has been tested for efficacy against SW620 colon cancer cells. Anti-NK1.1 on liposomes promoted the conjugation of liposomes to NK1.1-expressing NK cells to form “super” natural killer cells. Subcutaneously injected SW620 cancer cells led to the formation of initial metastases after 2–3 weeks that sequentially progressed into macroscopic metastasis during 3–6 weeks. TRAIL/anti-NK1.1-treated animal models demonstrated a significant suppression in the metastasizing properties of the primary tumors to the inguinal lymph nodes. Importantly, lymph nodes from control groups provided evidence of the presence of cancer cells in the peripheral zone of the tissues. Moreover, interior sides of the tissues were noticed to be infiltrated with clusters, glands, and sheets of neoplastic cells surrounded by fibrous tissues and few lymphoid cells. Furthermore, inguinal lymph nodes were replaced completely by tumors with a negligible fraction of lymphoid follicles within the tissue sections. Whereas lymph nodes did not show enlargement or infiltration of the cells from the primary tumors in the mouse models treated with TRAIL/anti-NK1.1 liposomes. Liposomal injections did not show signs and symptoms of liver toxicity. Mice injected with TRAIL/anti-NK1.1 liposomes demonstrated mild and non-significant alteration in the serum levels of liver enzymes [35].

These promising TRAIL formulations not only retained the pro-apoptotic functions of TRAIL but efficiently induced apoptotic death in Jurkat cells and mutant cells resistant to soluble recombinant TRAIL and chemotherapeutics. LUV-TRAIL induced activation of effector caspases proficiently and potently as compared to sTRAIL. LUV-TRAIL was found to be cytotoxic only against primary human leukemic cells but did not harm normal human lymphocytes [36].

Formation of high-order TRAIL oligomers in TRAIL-coated lipid nanoparticles enhanced DR5 cross-linking and increased apoptotic signaling. Lipid nanoparticles decorated with sTRAIL (LUV-TRAIL) effectively impaired the growth of HCT-116-derived tumors. It is important to mention that the soluble version of TRAIL used in these studies is comparable to the versions used in different phases of clinical trials (Dulanermin^®^) [37]. LUV-TRAIL is more cytotoxic as compared to sTRAIL in TRAIL-resistant NSCLC cell lines [38].

TRAIL is conjugated to magnetic ferric oxide nanoparticles by the attachment of primary amino groups of TRAIL to activated double bonds on the surface of nanoparticles. Fluorescently labeled TRAIL-conjugated nanoparticles were implanted in the ipsilateral hemisphere 7 days after the intracranial implantation of U251 glioblastoma cells. These TRAIL-conjugated nanoparticles considerably impaired the growth of the tumors derived from U251 glioblastoma cells [39].

Polymeric ultrasound contrast agents (UCA) (microencapsulated gas bubbles) can be fragmented into nanoparticles by focused ultrasound. TRAIL-conjugated UCA efficiently induced apoptosis in OVCAR-3 and A2058 cancer cells [40].

**Figure 4:**
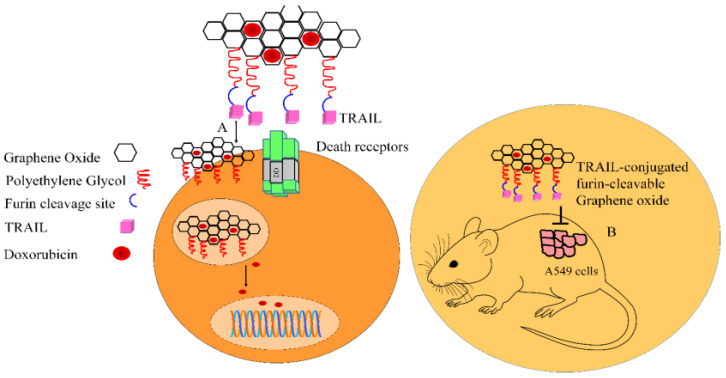
Graphene oxide-based nanocarriers delivered TRAIL and induced apoptotic death in cancer cells and shrinkage of the tumors.

Nanocarriers designed with graphene oxide, polyethylene glycol linkers, and a furin-cleavable peptide have been reported to effectively deliver TRAIL to the target sites. Graphene oxide-based nanocarriers accumulate in the tumor sites by enhanced permeability and retention (EPR) effects after intravenous administration. Plasma membrane-located furin proteolytically cleaves the peptide linkers, thus causing the release of TRAIL for interaction with receptors present on the surface of cancer cells. The graphene oxide nanosheet carrying doxorubicin is internalized into the cancer cells. In the endosomes, the release of doxorubicin is promoted, and consequently, intracellularly released doxorubicin accumulates in the nucleus to produce DNA damage. Intravenously administered TRAIL-conjugated furin-cleavable graphene oxide induced regression of the tumors in mice inoculated with A549 cells (Figure 4) [41].

Inhalable nanoparticles composed of human serum albumin (HSA) conjugated with doxorubicin and octyl aldehyde and adsorbed with TRAIL have been reported to be highly promising as potential nano-formulations for the delivery of TRAIL and chemotherapeutic drugs. TRAIL/Dox HSA nanoparticles effectively impaired the growth of the tumors derived from H226 cells in tumor-bearing mice [42].

TRAIL-functionalized liposomes mimicked membrane-displayed TRAIL. These liposomes efficiently interacted with DR4 and DR5 and potently induced apoptotic death. These liposomes were combined with anti-EGFR single-chain Fv fragments for targeted delivery of the payload to EGFR-positive cancer cells. Intravenously injected functionalized liposomes and intraperitoneally injected bortezomib led to shrinkage of the tumor xenografts in mice inoculated with Colo205 cancer cells [43].

HSA is an ideal delivery agent because of its remarkable properties. HSA is stable, non-toxic, non-immunogenic, biodegradable, and biocompatible. Intravenously administered TRAIL and doxorubicin-loaded HSA nanoparticles greatly suppressed tumor growth in mice subcutaneously inoculated with HCT116 cells [44].

PEGylation is classically viewed as a gold standard for extension of half-life of the biological molecules. Therefore, long-acting PEGylated TRAIL (TRAIL_PEG_) had an extended half-life, and it induced shrinkage of the TRAIL-sensitive palpable tumors. However, TRAIL_PEG_ did not induce apoptotic death in TRAIL-resistant tumors. Use of doxorubicin with TRAIL_PEG_ accelerated the activation of caspase-8 and caspase-3 through upregulation of DR5 in HT-29 cells. Doxorubicin-encapsulated hyaluronic acid-based nanoparticles worked synergistically with TRAIL_PEG_. Intravenous injections of HAC/DOX/TRAILPEG significantly impaired tumor growth in mice inoculated with HT-29 cells [45].

Oxaliplatin immunohybrid nanoparticles (OIHNPs) were designed for the delivery of oxaliplatin and anti-TRAIL as a promising strategy to treat colorectal cancers. Importantly, OIHNPs were not reported to be internalized in TRAIL-negative MCF-7 cells. Whereas cellular uptake of immuno hybrid nanoparticles was enhanced because of the receptor-induced endocytic process in TRAIL receptor-overexpressing HT-29 cells. OIHNPs inhibited the growth of the tumor xenografts in BALB/c nude mice subcutaneously inoculated with HT-29 cells [46].

Nanoparticle-engineered TRAIL-overexpressing adipose-derived stem cells have been shown to demonstrate important anticancer effects. Implantation of hADSCs into the hemisphere contralateral to the tumor site or injections via tail vein into the tumor-bearing mice was found to be effective. It was shown that inoculated hADSCs in the hemisphere contralateral to the tumor migrated towards the tumor mass along the corpus callosum post-implantation (10th day). However, hADSCs remained within the injection sites in the healthy brain. hADSCs displayed a potent migratory ability and glioma tropism in xenografted mice and presented remarkable capability to “seek” infiltrating glioma cells. Importantly, injection of hADSCs through a tail vein did not promote the distribution and detection of hADSCs in the brain. These findings indicated that local delivery within the brain was necessary for effective targeting of the tumors. Repetitive intra-tumoral injections of NPs/pTRAIL-hADSCs in patient-derived GBM mice led to an increase in the survival rate as compared to a single intra-tumoral injection [47].

**Figure 5:**
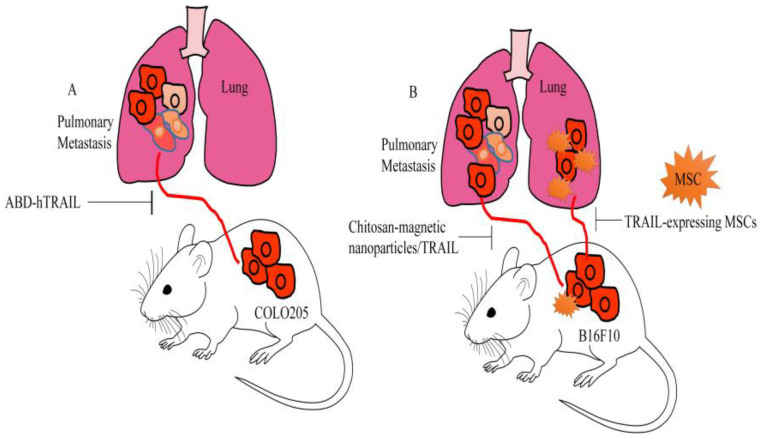
(**A**) Fusion of albumin-binding domain to hTRAIL increases circulatory half-life. Intravenously injected ABD-hTRAIL effectively inhibited the metastasizing abilities of COLO205 cancer cells. (**B**) Chitosan-modified iron oxide-based magnetic nanoparticles delivered TRAIL gene and inhibited metastatic spread of B16F10 melanoma cells. Intravenously injected TRAIL-transfected MSCs chemotactically migrated towards the lungs and severely hampered metastatic spread of B16F10 melanoma cells.

Fusion of the albumin-binding domain (ABD) to hTRAIL led to an increase in circulatory half-life. Fusion of the ABD domain with the N-terminal of TRAIL increased the anti-metastatic effects of hTRAIL by 3–4 times as compared to the C-terminal. Intravenously injected ABD-hTRAIL caused regression of subcutaneous tumor xenografts in mice. Moreover, ABD-hTRAIL effectively inhibited the metastasizing abilities of COLO205 cancer cells. Importantly, pulmonary metastatic nodules were found to be significantly suppressed by ABD-hTRAIL in mice injected with COLO205 in the tail vein (Figure 5A) [48].

## 4. Delivery of TRAIL Gene as PAYLOAD

Importantly, macrophages were engineered with mono-TRAIL and Tri-TRAIL, and the results revealed that Tri-TRAIL demonstrated higher cytotoxic effects against tumor cells as compared to Mono-TRAIL. Tail vein injection of Tri-TRAIL inhibited tumor growth in BALB/c mice subcutaneously inoculated with 4T1 cells [49]. Ionizable lipid nanoparticle-mediated delivery of TRAIL mRNA has been found to be efficacious against colon cancers [50].

Lipid nanoparticles (LNPs) potently protected nucleic acids from degradation and enhanced cellular uptake. TRAIL was fused with a receptor-binding domain (RBD) from the SARS-CoV-2 spike protein, encapsulated in LNPs to target colon cancer cells. Intra-tumorally injected LNP-encapsulated RBD-TRAIL efficiently inhibited the tumor growth in NOD-SCID mice models inoculated with resected tumor samples from colon cancer patients [51].

TRAIL-expressing plasmid was incorporated into cationic albumin-conjugated pegylated nanoparticles (CBSA-NP) were used against glioblastoma cells. CBSA-NP-hTRAIL selectively binds to negatively charged glycoproteins in the capillary walls of the brain. In animal models, negatively charged glycoproteins are located in brain vasculature. It has been shown that brain tumors expressed high levels of negatively charged glycoproteins. These glycoproteins are expressed not only in tumor cells but also in tumor vasculature. Wide distribution of CBSA-NP-hTRAIL was found in tumors. Expression of hTRAIL mRNA was detected throughout the periventricular region and cerebral cortex as well as in tumor tissues at 24 h after intravenously administered CBSA-NP-hTRAIL. Intravenously injected CBSA-NP-hTRAIL impaired tumor growth in mice intracranially implanted with C6 cells into the right striatum [52].

Polyplexes formed between DNA and cationic polymers are comparatively more stable than lipoplexes formed between cationic lipids and DNA. Biodegradable poly(amine-co-ester) terpolymers were used for the delivery of the TRAIL gene. Importantly, tail vein injections of coated TRAIL polyplexes induced retrogression of the tumor mass in mice subcutaneously inoculated with A549 cancer cells. The size of tumors developed from A549 cancer cells was ~300 mm^3^ in mice injected with coated TRAIL polyplexes as compared to the size of the tumors (~700 mm^3^) in mice administered with control polyplexes [53].

Angiopep-2 is a potential ligand that binds to LRP1 (low-density lipoprotein receptor-related protein-1) expressed on BCECs and glial cells and traverses the blood–brain barrier. Angiopep-2 has a high BBB transcytosis capacity. These hallmark features have advocated the use of Angiopep-2 as a targeting ligand. Different nanostructures have been functionalized with Angiopep-2 to direct therapeutic agents to the brain. There is a gradual increase in the clinical trials related to novel conjugates of Angiopep-2 (GRN1005, ANG1005) [54,55,56].

High-branching dendrimer polyamidoamine (PAMAM) has been tested for efficacy as a delivery vehicle. Importantly, a highly proficient gene delivery system was engineered by conjugation of Angiopep-2 to PAMAM using bifunctional PEG and formation of a complex with DNA. Cellular uptake mechanisms were investigated in glial cells, and findings suggested that DNA of PAMAM-PEG-Angiopep/DNA nanoparticles moved into the nuclei through the endosomal/lysosomal pathway. Essentially, PAMAM-PEG-Angiopep/pORF-TRAIL nanoparticles were injected into the tail vein of mice and impaired the tumor growth in mice orthotopically implanted with C6 glioma cells into the right striatum [57].

Second-generation TRAIL-receptor agonists have been designed to improve in vivo half-lives. Fc-scTRAIL belongs to the second-generation TRAIL-receptor agonist. It is produced by fusion of a single-chain TRAIL (scTRAIL) trimer to the Fc region of an IgG, resulting in the formation of a hexavalent TRAIL-receptor agonist. Fusion of Angiopep-2 to hexavalently engineered TRAIL demonstrated potent anticancer effects [58].

Transferrin (Tf)-modified polyamidoamine dendrimer (PAMAM) was prepared for the delivery of the TRAIL gene to the target sites. PAMAM-PEG-Tf/TRAIL effectively impaired tumors in mice implanted with C6 cells into the right striatum. Importantly, median survival time of PAMAM-PEG-Tf/TRAIL-treated rats was reported to be extended [59].

Positively charged amino groups on PAMAM surfaces have been reported to limit their value for clinical applications. Positively charged amino groups on the surface of PAMAM dendrimers interact with negatively charged biological lipid bilayer membranes [60]. PAMAM dendrimers cause hemolytic effects by the interaction of red blood cells with free cationic terminal amine groups on the surface of the dendrimers. Neutralization of the cationic groups of PAMAM by anionic or neutral functional groups led to prevention of the electrostatic interactions of PAMAM dendrimers [61,62].

There was a stable retention of PEGylated dendrimers in the blood pool of mice for at least 1 h, while non-PEGylated dendrimers demonstrated a high excretion rate through the kidney [63].

In another study, fifth-generation poly(propylene imine) dendrimers were tested for hemolytic toxicity and hematological parameters. Modification of dendrimers led to a significant reduction in the hemolytic activity and hematological parameters [64].

Modifications of PAMAM dendrimers by alkylcarboxylate chains, PEG, and cholesteryl chloroformate are also valuable for the delivery of plasmid TRAIL in BALB/c mice inoculated subcutaneously with C26 colon cancer cells. Modified PAMAM dendrimers did not cause animal model toxicity and histopathological changes in organs [65].

Tyrosine kinase receptor 3 ligand (FL) and TRAIL have been reported to synergistically inhibit cancer progression. Gene-carrying cationic nano-liposomes were transfected into dendritic cells. Simultaneously injected FL-expressing dendritic cells and TRAIL-expressing dendritic cells induced shrinkage of the tumor xenografts in mice inoculated with Lovo cells [66].

Magnetic core–shell nanoparticles delivered and activated a heat-inducible gene vector that encoded TRAIL in adipose-derived mesenchymal stem cells (AD-MSCs). TRAIL-expressing AD-MSCs potently inhibited in mice intraperitoneally injected with ovarian A2780 cancer cells [67].

Dimerization of HIV-1 TAT peptides played a significant role in the delivery of therapeutic genes. Administration of dTAT/pTRAIL nanoparticles via intra-tracheal spray caused shrinkage of the tumors in mice inoculated with LLC cells. dTAT/pTRAIL considerably reduced the macroscopic lung tumor multiplicity [68].

Triazine-modified dendrimer G5-DAT66 is a valuable delivery vehicle for the TRAIL gene. G5-DAT66/pTRAIL induced slight alteration in body weight and mild systemic toxicity of G5-DAT66. Additionally, G5-DAT66 demonstrated extremely low hemolytic activities. Intra-tumorally injected G5-DAT66/pTRAIL led to efficient inhibition of tumor growth in the MG-63 tumor xenograft model [69].

Chitosan-modified iron oxide-based magnetic nanoparticles acted as a promising delivery vehicle for the TRAIL gene. These iron oxide-based magnetic nanoparticles considerably impaired the metastatic dissemination of melanoma B16F10 cells to the surface of the lungs in rodent models of metastasis (Figure 5B) [70].

Positively charged polymer PEI-modified Fe_3_O_4_ magnetic nanoparticles have previously been analyzed for the transfer of gene vectors in the presence of a magnetic field. Tumor-specific promoter hTERT was used to regulate the expression of the TRAIL gene in tumor tissues. TERT-TRAIL magnetic complexes were injected into the tumors derived from Tca83 cancer cells. Importantly, mean tumor volumes were 420 mm^3^ for animal models treated with DNA magnetic complexes and 864 mm^3^ for the control groups [71].

PTEN and TRAIL genes were loaded into zein nanoparticles (ZNPs) for an analysis of efficacy in tumor-bearing mice. PTEN- and TRAIL-loaded zein nanoparticles induced suppression of MMP-2 and VEGF in liver tissues of mice inoculated with HepG2 cells [72].

DOTAP-like cationic lipid was designed by changing the trimethylammonium of DOTAP to biguanide. The biguanide group of DOBP was functionally similar to metformin in action and exerted anticancer effects by activation of the AMPK pathway. Biguanide, DOBP-encapsulated TRAIL plasmids into lipid-protamine-DNA (LPD) nanoparticles demonstrated promising results. Tail vein injections of DOBP-LPD-TRAIL nanoparticles exhibited superior efficacy and induced tumor regression in mice inoculated with H460 cells [73].

## 5. Polyethyleneimine as a Delivery Vehicle for TRAIL

PEI is one of the most studied cationic DNA transfection reagents. However, b-PEIs with certain molecular weights at specific concentrations lead to RBC aggregation and lysis [74]. Intravenously injected PEI caused severe acute damage and induced rapid animal death within hours [75].

Modifications of branched polyethyleneimine (b-PEI) using mesquite gum have been experimentally investigated for hemotoxicity. Therefore, carboxymethylated mesquite gum-grafted b-PEI copolymers demonstrated minimal hemotoxicity (2% at 0.03 and 0.01 µg/mL), making it a non-hemolytic material at those concentrations [76].

Twenty-five kilodalton b-PEI/pDNA complexes with polyacrylic acid exhibited slightly lowered surface charge, ~31 mV (without PAA) to ~21 mV (with PAA). Thus, the favorable effects of lipid-substituted LMW b-PEIs on hemolysis are mainly because of a decrease in the surface charges [77].

Polyethyleneimine (PEI) is widely used in gene delivery systems because of its characteristically unique ability to build a complex with DNA and support the release from endosomes through “proton sponge effects”. TRAIL expression was higher in the large PEI/pTRAIL^high^ NPs group. Consequently, PEI/pTRAIL^high^ nanoparticles showed superior efficacy against HeLa cells as compared to PEI/pTRAIL^low^ nanoparticles. Furthermore, TRAIL expression in the tumors derived from HeLa cells was high in the mice treated with PEI/DNA^high^ nanoparticles as compared to the PEI/DNA^low^ NP-treated groups. The large nanoparticles enter into the tumor cells primarily through the macropinocytosis pathway. These nanoparticles proficiently dissociate in the cytoplasm and release cargo DNA [78].

Cell-specific targeting molecule folate (FA) is linked to polyethylene glycol (PEG). FA-PEG is engrafted onto the hyper-branched PEI. FA-PEG-grafted-hyperbranched-PEI (FA-PEG-PEI) induced effective condensation of plasmid DNA (pDNA) into nanoparticles. Cytosine deaminase (CD) is an enzyme that deaminates the prodrug 5-fluorocytosine (5-FC) and converts it into the highly cytotoxic 5-fluorouracil (5-FU). TRAIL and CD-loaded-FA-PEG-PE induced retrogression of the tumor mass in mice implanted stereotactically with C6 glioma cells into the right caudate nucleus of Wistar rats [79].

In another study, cyclic RGD peptides have been tested for biomedical applications for targeting the tumor-associated vasculature. RGD-PEG-PEI is used as a non-viral gene carrier for targeted therapy of glioblastoma cells. Cyclic RGD peptides bind to integrin α(v)β(3)-over-expressing U87 glioblastoma cells and neovasculature. Thus, co-delivery of brain-targeted CDX-PEG-PLA-PTX micelles led to a dramatic increase in the gene transfection efficiency in the intracranial brain tumors. RGD-PEG-PEI/pORF-hTRAIL nanoparticles in combination with paclitaxel-loaded CDX-PEG-PLA micelles were found to be effective in rodent models of glioblastoma. Importantly, the median survival was found to be significantly longer for intracranial glioblastoma-bearing animal models combinatorially treated with TRAIL and paclitaxel. However, median survival was reduced in mice treated with CDX-PEG-PLA-PTX (25.5 days) or RGD-PEG-PEI/pORF-hTRAIL (24.5 days) [80].

Linear poly(ethylenimine) (lPEI) was engrafted onto the block copolymers of poly(l-lysine) (PLL) and PEG, yielding a ternary copolymer, PEG-b-PLL-g-lPEI (PPI), for an efficient delivery of the TRAIL gene. These delivery vehicles demonstrated mild cationic toxicity as well as significantly extended circulation time in tumor-bearing mice. With folate as a classical targeting ligand, the FA-PPI/pDNA complexes demonstrated remarkably higher transgene activity in folate receptor (FR)-overexpressing cancer cells. Importantly, FA-PPI-mediated effective transfection of the TRAIL gene in human hepatoma Bel 7402 cells potently induced apoptotic death [81].

Peptide-mediated targeting of tumors has started to gain appreciation as an innovative strategy for cancer therapy. Therefore, D-SP5 peptide-modified, highly branched polyethylenimine (D-SP5-PEG-PEI) was used for delivery of the TRAIL gene. D-SP5-PEG-PEI/pTRAIL effectively caused shrinkage of tumors derived from gastric adenocarcinoma SGC7901 cells in nude mice [82].

Sandwich-type layered (PEI)-coated gold nanocomposite outerlaid with a nucleus-targeted dexamethasone (Dexa), Au-PEI/DNA/PEI-Dexa nanocomplex has been used as a delivery system for TRAIL. Administration of 50 µL of Au-PEI/pTRAIL/PEI-Dexa polyplexes every five days impaired the growth and size of the tumors in mice inoculated with Hep3B cancer cells [83].

Biodegradable diblock copolymer TPGS-b-(PCL-ran-PGA) nanoparticles modified with a polyplexed PEI efficiently deliver TRAIL to the target cancer cells. Importantly, mean survival time was found to be significantly longer in mice models treated with TRAIL/endostatin-loaded nanoparticles [84].

PEI600-Cyd is prepared by a cationic polymer composed of LMW PEI with a molecular weight of 600 Da cross-linked by β-cyclodextrin (β-CD). PEI(600)-Cyd has the ability to transfect TRAIL to MSCs. Intravenously injected TRAIL-transfected MSCs chemotactically migrated towards the lungs 1 day after inoculation in a rodent model. Consequently, after 7 days, MSCs were found to be detected in the lung area. TRAIL-expressing MSCs significantly inhibited the pulmonary metastatic spread of B16F10 melanoma cells in rodent models (Figure 5B) [85,86].

Targeted iron oxide nanoparticles coated with chitosan-PEG-PEI copolymers and chlorotoxin (CTX) were used for the delivery of TRAIL. Chlorotoxin was attached to the nanoparticles to improve targeted transfection efficiency. T98G-bearing mice treated with NP-TRAIL-CTX demonstrated rapid regression in the size and volume of the tumors [87].

Radionuclide iodine–131-labeled chlorotoxin (131I–TM601) is currently being tested for efficacy for the treatment of malignant gliomas [88]. Overall, chlorotoxin peptides have notable applications, and the conjugation of chlorotoxin peptides to anticancer drugs will be valuable, particularly for the targeting of glioblastoma.

## 6. Nanotubes for Delivery of TRAIL

Single-walled carbon nanotubes (SWCNTs) have emerged as a promising candidate at the crossroads between a proof-of-principle concept and a significant delivery vehicle. TRAIL-functionalized nanotubes had been shown to demonstrate superior efficacy as compared to TRAIL. Essentially, functionalization of TRAIL on SWCNT mimicked membrane-bound TRAIL and favored higher-order receptor aggregation, leading to better activation of caspase [89]. These findings are exciting and need to be tested in xenografted mice. Therefore, it will be interesting to analyze if TRAIL-functionalized nanotubes led to regression of palpable tumors in mice subcutaneously or orthotopically inoculated with cancer cells.

1-pyrenebutyric acid N-hydroxysuccinimide ester functionalized boron nitride nanotubes (BNNTs) were utilized to anchor the TRAIL protein. BNNTs were mixed with methoxy-poly(ethylene-glycol)-1,2-distearoyl-sn-glycero-3-phosphoethanolamine-N-conjugates for a better dispersion of these TRAIL nanoparticles in aqueous solution. Overall, nano-vectorization of TRAIL with BNNTs potently improved its binding affinity for DR4 and DR5 at 37 °C [90].

Structurally engineered anodic alumina nanotubes are exceptional delivery vehicles for TRAIL. AANTs were internalized readily by macrophages and breast cancer cells. It is relevant to mention that around 40% of TRAIL gets released after 30 min, followed by a sustained release pattern extended for 240 min. Burst release was not observed under these conditions [91]. Collectively, these findings indicated that unique lumen structures and narrow-open ends of AANTs made them valuable nano-reservoirs for a sustained release of the payload and to attain ultra-high loading of TRAIL molecules.

## 7. Concluding Remarks

Doubtlessly, interdisciplinary researchers have revolutionized platform technologies to produce biologics from living systems. Next-generation nanotechnological advancements and materials science have opened new horizons in formulation chemistry and the administration of pharmaceuticals.

The field of nanomedicine has undergone remarkable broadening, and proof-of-principle studies have catalyzed the design and development of clinically effective nanoparticles.

Delivery of TRAIL-based therapeutics to the tumor microenvironment is a rapidly evolving field and needs extensive research. There is a continuous need to target the tumor microenvironment by combinatorial strategies consisting of chemotherapeutic drugs and TRAIL, TRAIL and antagonistic antibodies against PD-1 for efficient immunological activation within the tumor microenvironment in xenografted mice. Similarly, multidimensional approaches to improve and enhance the delivery of TRAIL-based therapeutics will open new horizons to reap maximum benefits of nanotechnological strategies. 

Surface decoration of nanoparticles with TRAIL or death receptor-targeting agonistic antibodies is a promising strategy for efficient killing of cancer cells in tumor-bearing mice (Table 1). However, these strategies have to be critically analyzed in advanced animal model studies, specifically in the context of pharmacokinetic and pharmacodynamic studies, to overcome the challenge of attrition rate.

Ground-breaking advancements in nanoparticle design have empowered researchers to overcome heterogeneous and multifaceted barriers for the delivery of TRAIL-based therapeutics. Therefore, intelligently designed nanoparticles can enhance efficiency in general delivery applications while enabling tailored designs for precision applications. Overall, these findings are intriguing, and rationally designed clinical trials will provide a better evaluation of the therapeutic efficacy of nanomedicine associated with TRAIL-based therapeutics.

## Figures and Tables

**Table 1:** Nanoparticle-mediated delivery of TRAIL for the inhibition of tumor growth in rodent models.

Type of Nanoparticles	Cancer Type and Tumor Shrinkage	Reference
TRAIL-ODN-PLGA-NPs	MDA-MB-231 Tumor volume (644.9 ± 162.1 mm^3^) Tumor Weight (1218.0 ± 178.5 mg)	[25]
Mitoxantrone Combined with Engineered TRAIL-Nanovesicles	Orthotopic 4T1 tumor (79.4%)	[26]
TRAIL-PEG-NPs	HCT-116 (78.5%)	[33]
Doxorubicin-bound albumin nanoparticles containing a TRAIL Protein	HCT116 Tumor volumes (933.4 mm^3^) Tumor Weight (0.60 ± 0.18 g)	[44]
ABD-hTRAIL	COLO205 Tumor volumes 55 ± 10.4 mm^3^ Tumor masses 0.06 ± 0.01 g	[48]
Coated TRAIL polyplexes	A549 300 mm^3^	[53]
PEI-modified Iron oxide nanoparticles loaded with TRAIL mRNA	Tca83 420 mm^3^	[71]

## Abbreviations

TNF	(Tumor necrosis factor)
FasL (CD95L)	(Fas ligand)
TRAIL	(TNF-related apoptosis-inducing ligand)
DR4/DR5	(Death receptor 4/death receptor 5)
DISC	(Death-inducible signaling complex)
FADD	(FAS-associated protein with death domain)
XIAP	(X-LINKED inhibitor of apoptosis)
SMAC	(Second mitochondria-derived activator of caspase)
RAPTOR	(Regulatory associated protein of mTOR)
CTL	(Cytotoxic T lymphocytes)
ERCC	(Excision repair, complementing defective, in Chinese Hamster-2)
DOTAP	(1,2-dioleoyl-3-trimethylammonium propane)
CBSA-NP	(Cationic albumin-conjugated pegylated nanoparticles)
LRP1	(low-density lipoprotein receptor-related protein-1)
PAMAM	(Polyamidoamine dendrimer)
PTEN	(Phosphatase and tensin homolog)
VEGF	(Vascular endothelial growth factor)
MMP2	(Matrix metalloproteinase)
